# The Transmission of Tuberculosis by Insufficiently Cooked Bread

**Published:** 1907-09

**Authors:** 


Translated for the Texas Medical Journal.

### The Transmission of Tuberculosis by Insufficiently Cooked Bread.

The possibility of the transmission of tuberculosis by means of insufficiently cooked bread has been discussed by Dr. A. Lesalle and Dr. Barney, and they have arrived at the positive conclusion that dough kneaded by careless workmen suffering from tuberculosis can be the vehicle for conveying the bacillus of Koch and that insufficient cooking does not destroy the vitality of these germs.

Dr. C. Chicote, of Madrid, has also experimented along these lines and has reached the same conclusions and has proven by careful experiments that the disease can be spread in this manner. In five experiments made by him the sputum of a tuberculous patient was mixed with dough and cooked (insufficiently) in the same manner that the bread was cooked, same oven and temperature and upon examination afterwards, two out of five samples of dough thus treated were found to have the bacillus of Koch still alive. It appears that in Madrid bread is made with a larger portion of water than is really necessary, and this dough being purposely undercooked so as to retain a part of the moisture (in order to keep up the weight) bread so prepared by tuberculous workmen is highly dangerous.

To avoid this danger Dr. Chicote recommends that, first, baking bread by hand be absolutely prohibited; second, that a permanent medical inspection of all bakers be maintained and whenever any of them are found to be tubercular they be prevented from working in the bakeries.—*Translated from the Bulletin of the Superior Board of Health of San Salvador, Dr. Rafael V. Castro, Director.*

---

PUBLIC HEALTH LABORATORIES

(CHEMICAL AND BACTERIOLOGICAL.)

LONDON HOSPITAL MEDICAL COLLEGE, LONDON, E.,

AND CHELMSFORD.

February 1, 1907.

Results obtained upon analysis of a sample of Meatox, prepared by Charles Marchand, of New York, and submitted by Messrs. Lamont Corliss & Co., January 10, 1907, contained in hermetically sealed bottles.

The material submitted was in a granular condition, of yellowish color, faint meaty odor and of agreeable flavor.

After digesting in water the material became soft, and when placed under the microscope it appeared to consist of muscular fibre more or less altered by the action of heat. Tests were made for detecting the presence of preservatives, but no indications were obtained of their presence. Even the amount of salt present was under one per cent.

The chemical analysis gave the following results:

| | | |
|---|---|---|
| Moisture | 5 | per cent. |
| Fat | 12.1 | per cent. |
| Assimilable proteid | 73.8 | per cent. |
| Extractive matter (less gelatine) | 7.7 | per cent. |
| Ash | 2.05 | per cent. |

The fat was examined and corresponded in character with the fat from oxen.

The preparation no doubt contains all the constituents of beef in a very dry and, therefore, stable and concentrated condition. It is about five times the strength of fresh beef.

JOHN C. TRESH.

*P. S. Remarks.*—The extractives in our case include creatin, etc., which is nitrogenous, though not proteid. This extractive gave us the reactions of beef extract. The preparation should be a very useful one and supersede to some extent meat extracts, since the latter are merely stimulants whilst the former is both stimulant and nutritive.

Some one is sure to raise the question about the lowness of the "ash," and I should like to know Prof. Marchand's explanation.

Yours truly,

J. C. T.

*N. B.*—The cause of the small percentage of ash is that I purposely eliminate as much of the mineral substances as possible from the raw meat, so as to increase the percentage of proteid.

CHARLES MARCHAND.

